# The Layered Syntactic Structure of the Complementizer System: Functional Heads and Multiple Movements in the Early Left-Periphery. A Corpus Study on Italian

**DOI:** 10.3389/fpsyg.2021.627841

**Published:** 2021-04-27

**Authors:** Vincenzo Moscati, Luigi Rizzi

**Affiliations:** ^1^Department of Social, Political and Cognive Science, University of Siena, Siena, Italy; ^2^Chair of General Linguistics, Collège de France, Paris, France

**Keywords:** complementation, syntax & grammar, syntactic movement, corpus linguistic analysis, language acquisition, grammatical dependencies

## Abstract

In this paper we document the developmental trajectory of the complementizer system (CP-system) in Italian by looking at the earliest spontaneous production of eleven young children, whose transcriptions are available on CHILDES. We conducted a novel corpus analysis, tracking down a number of constructions in which the clausal left-periphery is activated. First, we considered the appearance of the different complementizer particles in the CP-system, which overtly realize the three distinct functional projections ForceP, IntP, and FinP. The analysis revealed that children acquiring Italian correctly use these complementizer particles already in the third year of life. Second, we looked for the simultaneous activation of different functional projections within the CP-system. We went through our corpus searching for complex sentences in which more than one constituent was moved to the left periphery. This option is allowed by the adult grammar of Italian and, as our search revealed, it is also attested in the grammar of young children. Soon after their second birthday, sequences in which a left-dislocated Topic and a Wh- element co-occur are attested, directly supporting the existence of a (high) Topic position above FocusP. Moreover, movement in general conforms to the constraints of the adult grammar, with no attested violation of obligatory inversion (a consequence of the Q-Criterion). Importantly, “*why*-questions” did not require inversion, much as in the adult grammar of Italian. Taken together, children's use of complementizer particles and their activation of multiple landing sites for movement show that 2-year-olds already possess a richly articulated functional structure of the CP-system, aligned to the layered adult structure. In concluding the paper, we also discuss some temporal differences between constructions activating high and low portions of the CP-system. In particular, we detect a temporal precedence for wh-questions over why-questions. Since the former activate a lower projection, this is consistent with the recently proposed *Growing Trees* hypothesis, according to which the development of the CP-system proceeds stepwise.

## Introduction

Looking at children's spontaneous productions, a quick albeit gradual development can be easily observed in the morpho-syntactic complexity of their early sentences. Between the 2nd and the 3rd year, moving from the very first constructions in the two-word stage, children steadily advance through more articulated sequences that step-by-step converge on the adult grammar. Characterizing this process has been a major goal of research in language acquisition, a goal which necessarily calls for a constant interaction between developmental psychology and linguistic theory.

This exchange has proved to be useful in many ways, including the characterization of the early inflectional system. A telling example comes from much work, begun during the 90's, on the relation between verbal morphology and word-order. In language after language, it was found that from early on there is a tight link between the position of the verb and its inflection. Just to mention some observations, French-speaking children systematically vary the verb's placement depending on finiteness, so to match the adult distribution: Pierce (1992) showed that, whereas finite forms precede the negation marker *pas*, non-finite forms follow it. Similarly, a strong correlation between morphology and clausal position was also found in the productions of young German-speaking children, with the V2 position selectively used for finite verbs only (Verrips and Weissenborn, 1992; Poeppel and Wexler, 1993).

These lines of research were inspired by developments in linguistic theory which, shortly before, had offered a natural explanation of such correlations. From the seminal work of Pollock (1989), morphosyntactic features have been associated with independent syntactic projections, strictly ordered. Therefore, the syntactic features encoded in the verbal morphology can be checked through head-movement of the verb. This captures the observed link between word-order and inflection, both in adult and early grammars. Later on, Pollock's approach was developed and systematized in a line of research which eventually led to Cinque's (1999) comprehensive cartographic analysis of the structure of the IP.

In parallel, a richly structured hierarchical configuration has also been proposed to capture the higher portion of the clause. Just as the inflectional system can be seen as a multi-layered zone of ordered projections, so too the Complementizer system can be “split” into an articulated set of projections, each with its own well-defined properties. The gain has then been comparable with the Split-IP proposal, with advances on the word-order properties of the elements of the complementizer system, and on the study of the interfaces with sound and meaning of various left-peripheral constructions.

According to the Split-CP proposal initially presented in Rizzi (1997), the complementizer can be viewed as a syntactic space delimited by Force and Finiteness (Fin), including various positions dedicated to expressing particular scope-discourse properties: the scope of operators of different kinds (interrogative, relative, exclamative, etc.), discourse-related articulations such as topic–comment and focus–presupposition, the position occupied by highlighted adverbials, etc. All these projections must respect some ordering constraints, attested cross-linguistically, that can be captured by cartographic representations.

A layered CP-system offers a more articulated structure in comparison with traditional representations involving a single C-position. Much as the adoption of an articulated structure of the IP-system led to many fundamental observations on the properties of early clauses, the articulated structure of the complementizer has also the potential to reveal important characteristics of children's first utterances.

In this paper, our major goal is to document the development of the CP-system in Italian. A natural starting point in this direction is to consider the appearance of the various complementizer particles in children's first productions. Since they instantiate the heads of different left-peripheral projections, their occurrence provides an important landmark that could inform us about the initial skeleton of the CP in young children.

The structural positions of the Italian complementizer particles in the adult language can be illustrated by looking at their order in relation to topics, starting from the finite complementizer *che* (“that”). This element can be used to introduce both relative clauses and complement clauses and it arguably sits in ForceP, the highest position within the CP field, as confirmed by the fact that it can only precede (2a) but cannot be preceded by a topic (2b):


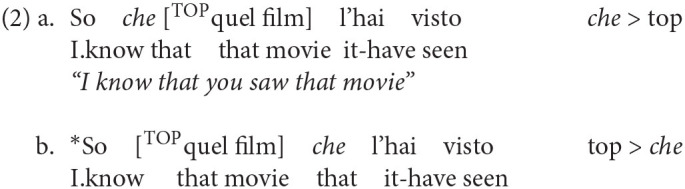


A lower structural position is instead occupied by the particle *se* (“if”). It is used to introduce conditional clauses and indirect yes/no interrogatives. It sits in an intermediate position within the CP-system and it can be either followed (3a) or preceded (3b) by a topicalized constituent:


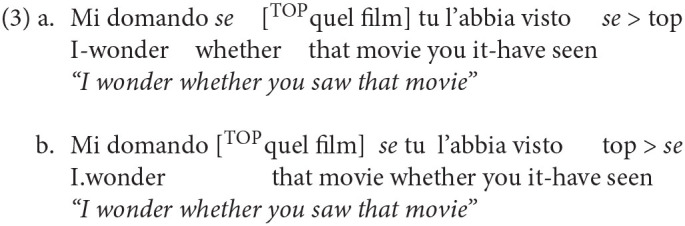


Finally, the last particle is the non-finite complementizer *di* (homophonous to preposition *di* “of”), which marks the low edge of the CP-system in control constructions. In this position, FinP, it can only be preceded by topics, as shown by the contrast in grammaticality between (4a) and (4b):


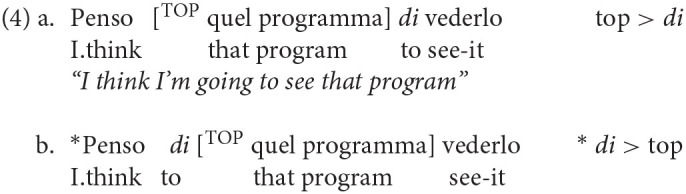


On the basis of these and other considerations (see Rizzi and Bocci, 2017 for an overview), the three particles can be considered as the heads of three distinct projections, as in the representation below:


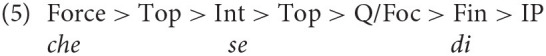


Representation (5) also includes the landing site of wh-movement, designated by Q/Foc. This notation captures the fact that in Italian main clauses wh-movement and left-peripheral focus movement compete for the same position (see Bocci et al., 2018 for discussion)^1^.

Currently, less is known about the emergence of these particles in early Italian and a corpus analysis could help us to determine if they all appear within the same temporal window or if, instead, there is a different timeline characterizing each one.

The potential of adopting a layered CP-structure was already appreciated in a corpus study reported in Mastropavlou and Tsimpli (2011), where the refined representation of the Greek CP proposed in Roussou (2000) was combined with a typology of grammatical traits (Tsimpli and Stavrakaki, 1999) distinguishing between interpretable and uninterpretable features in the sense of Chomsky (1995). Mastropavlou and Tsimpli were primarily interested in the emergence of complementation in children with Developmental Language Disorder. However, data from a control group of Typically Developing (TD) children was also discussed. Spontaneous production for the TD controls covered a single time-window above age 3, spanning over a short 2-months interval. By looking at this brief interval, Mastropavlou and Tsimpli showed that, by and large, TD children were already able to use the complete array of complementizers found in the adult language. This led to the conclusion that 3-year-olds can correctly use and alternate the various CP particles, in accordance with their appropriate grammatical function.

In view of this result and in order to document the gradual appearance of the complementizer particles while it might still be in progress, we believe it will be instructive to closely focus on an earlier period. Our corpus analysis will therefore be based on a longitudinal corpus with regular samples taken between age 1;5 and 3;5.

In this time-window, soon after age 2, children's first forms of adult-like embedding are documented, perhaps preceded by a short preparatory stage in which sequences of clauses resemble a matrix/embedded relation without an overt complementizer (e.g., “*do you see”* “*he is playing*”). This “*preconjunctional stage”* (Penner and Mueller, 1992) has been occasionally reported across languages (Hebrew, Armon-Lotem, 1997; Italian, Cipriani et al., 1998; Greek, Mastropavlou and Tsimpli, 2011). However, the status of these constructions remains elusive and they are also rapidly followed by the appearance of overt complementizers. We start from here, focusing on the first emergence of the full-fledged forms of embedding, introduced by overt particles. Moving on this firmer ground, we used a systematic semi-automated search to isolate all subordinate clauses in the spontaneous speech of 11 Italian-speaking children, to establish the timing in which the different types of embedding appear.

Looking at English, Bloom et al. (1980) and Bowerman (1979) have observed that complement clauses seem to precede adverbial and relative clauses. However, the inverse order has been reported in Swiss German by Penner (1995) and in Hebrew by Armon-Lotem (2005). It should be noticed, however, that if an advantage for relative clauses exists, it does not last long. Armon-Lotem, for example, reported the first occurrence of relatives in the Lior corpus at 2;1 followed by complement clauses only a month later at 2;2. In general, when the age of first-occurrence of the different types of subordination is considered, previous studies report a mixed pattern, with very brief time-differences that go in one direction or the other. As for Italian, there are no available data supporting a different course of acquisition for the two types of embedding. Interestingly, both relatives and complement clauses are introduced by the finite complementizer *che*. Thus, documenting the use of this particle could add some additional evidence in favor—-or against—-the idea that the two constructions develop at a different pace.

We also extend the corpus analysis in a second direction, looking at sentences with left-dislocated constituents that occupy the specifier of Top and the Q/Foc projections in the adult CP-system, represented in (5). To illustrate, consider the position of the direct object “*the match”* in the following sentences. In (6), a simple declarative sentence, the object occupies its canonical post-verbal position:


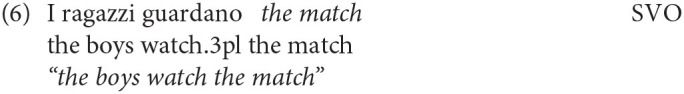


From its base position, the direct object could be moved to the Q/Foc projection, as in wh-questions (7) or in constructions expressing corrective focus (8):


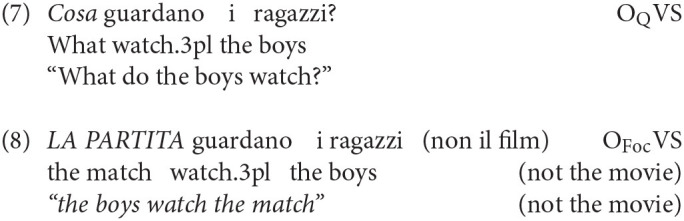


A further possibility is to dislocate the direct object to a Topic position, in this case also accompanying it with a co-referring clitic (9)


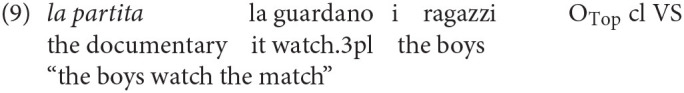


Since the different movement types illustrated in (7-8) and (9) trigger different syntactic positions, multiple movements to the left-periphery are also possible. For example, sentences (10) and (11) are perfectly grammatical in Italian: in both, a subject dislocated in a topic position precedes the direct object in Q/FocP, regardless of whether this latter is a wh-pronoun or a (correctively) focused DP:


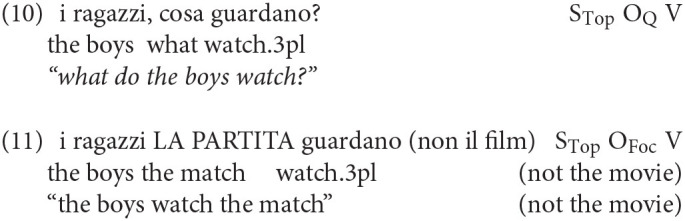


From the point of view of language acquisition, sentences like (10) or (11) would be very informative on the structure of the early CP. In fact, if attested, they would constitute the most direct evidence in favor of the emergence of a layered CP.

No example of the kind of (10) and (11) has ever been reported for Italian, but the importance of these constructions was already noticed in Soares (2006) in her corpus study on European Portuguese. Looking at the spontaneous production of three children, she found the occurrence of some sentences similar to (10), in which a left-dislocated topic preceded the wh-constituent. Although the majority of these examples were found in the speech of children already in their 4th year, a few were also found in younger children between 1;10 and 2;2. To date, however, the examples reported in Soares are still an isolated case and, to the best of our knowledge, other examples of multiple A'-movements in the early left-periphery have not been reported elsewhere in other Romance varieties.

Constructions of the kind in (11) with a pre-focal topic, are unattested in early spontaneous production. These structures have been experimentally investigated only in older children by Moscati et al. (2016) and only with respect to possibly ambiguous strings with two sentence-initial nominal constituents. The investigation of constructions like (11) can be hardly carried out by looking at the natural spontaneous production, since the use of the left-peripheral focus position is highly restricted in Standard Italian and it is only used in very special contexts, to express corrective and mirative focus (Bianchi et al., 2016). In light of this consideration, we do not expect the constructions in (11) to be attested in a natural production corpus of young children. We therefore will focus on examples similar to (10), with a wh- preceded by a left-dislocated topic. They are more likely to occur in spontaneous production, especially as they have been already observed in European Portuguese. We will then try to strengthen and possibly extend the initial observation made by Soares to Italian.

This issue of movement into the left-periphery also intertwines with other considerations about core cases of wh-movement. Following Rizzi (1996, 1997), Wh-movement in matrix clauses needs to satisfy an additional requirement that forces a local Spec-head relation between the wh-element and the inflected verb, a requirement called “the Wh-Criterion” in the reference quoted (and the Q-Criterion in later work). In that approach, the wh-element carrying the Q-feature must enter into a Spec-head relation with a verbal head sharing the same feature. This enforces I to C movement in questions. This requirement has the consequence that an overt subject cannot intervene between the wh-element and the inflected verb, as shown by (12): it must be post-verbal as in (13) or topicalized, as in the previous example in (10)


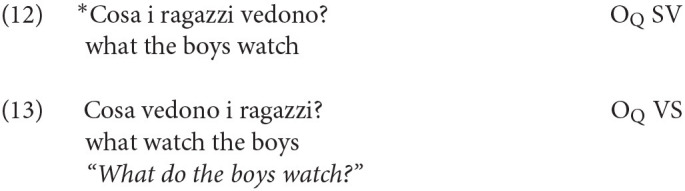


Turning to acquisition, the question arises whether children adhere to the Q-Criterion from early on, excluding the presence of an intervening constituent in general, and the subject in particular, between the wh- and the finite verb/auxiliary. In this respect, a further important refinement has to be made, since not all Wh-elements end up in the position requiring I-to-C movement. Other elements like *Perché* (Why) in matrix clauses are base generated in Spec/IntP, a head which presumably is inherently endowed with the feature +Q, hence the satisfaction of the Q-Criterion does not require movement of the inflected verb, so that the subject (or other material) can occur in between (see Rizzi, 2001 and much subsequent work for analysis of this pattern, and Thornton, 2008 for evidence that some children acquiring English go through an “Italian” stage, not requiring inversion with *why* questions). The grammaticality of both (14) and (15) with and without a preverbal subject illustrates this point:


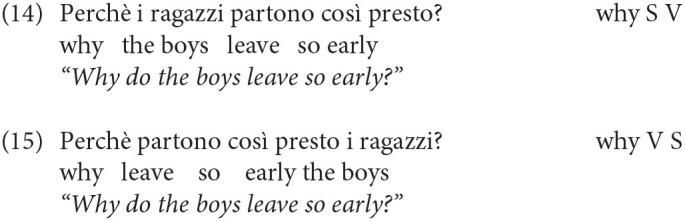


An asymmetry between (12–13) and (14–15) has been already documented in Guasti (2000) by analyzing the transcription of 5 Italian-speaking children. We will extend here the observation to a larger corpus, including 6 other children (Camilla, Rosa, Francesco, Elisa, Gregorio and Marco) and look for potential violations to the Q-Criterion. The absence of the ungrammatical construction in (12), together with the alternation in (14–15) would be telling about the early left-periphery: if children at age 2 already hypothesized its articulated structure with distinct positions for IntP and Q/FocP (we will continue to use this label to refer to the landing site of regular wh-movement), we predict that they will require inversion, but in a selective manner: inversion will be obligatorily found only for wh-constituents that sits in Q/Foc, but not in *why* questions.

## A Corpus Study

In the previous sections, we introduced a series of issues whose investigation could shed some light not only on the articulation of the CP-system in young speakers of Italian, but also more in general on the development of the higher functional spine of the clause. We believe that some of these questions can be addressed through a systematic analysis of spontaneous productions, on the basis of the corpus resources currently available.

As we pointed out in the introduction, a first description of the early CP must include particles that are used to convey one of its primary functions: clausal embedding. Summing up the previous discussion, a corpus analysis could help us answering the two following questions:


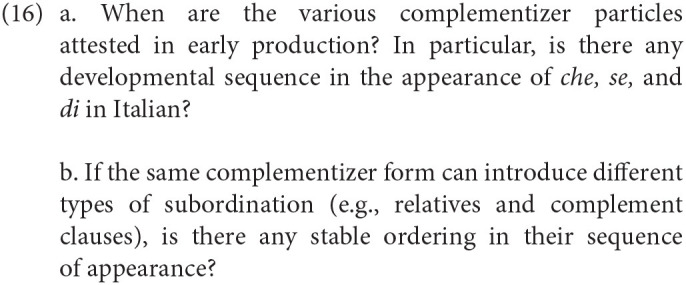


Turning to structures that would require movement of constituents into left-peripheral positions, they could add evidence in favor of a layered CP-system. In particular, we will look at whether the early CP provides a syntactic space with more than one position. If this is the case, we expect not only that multiple dislocations are possible, but also that I-to-C verb movement will obey the specific syntactic requirements enforced by different functional projections. This can be summed up with two additional research questions:


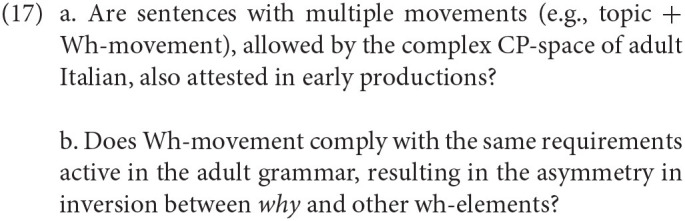


These questions will be framed within the functional hierarchy in (5) that describes the left-periphery of the target grammar^2^. Clearly, answering the questions in (16) and (17) would have implications for existing theories of clausal development. Considering these implications is outside the purpose of this paper, which is essentially descriptive. We will only limit the discussion, at the very end of the paper, to a very recent proposal presented in Friedmann et al. (2020) whose main innovation with respect to the precursors is the crucial use of the cartographic representation in (5).

In order to answer the questions in (16) and (17), we conducted a new corpus study looking at the transcriptions of the spontaneous productions of 11 Italian-speaking children available on CHILDES (MacWhinney, 2000) for a total of 128 files. Overall, our corpora cover a timespan that stretches from 1;4 (Francesco) to 3;4 (Camilla). Depending on the number of files and the age of the child, the absolute size varies considerably across the different corpora. A general summary of the properties of the individual corpora is provided in Table 1. The summary includes the source, the name of the child, the age of the child in the first and in the last file, the total number of files and the size of each corpus calculated in terms of the raw number of child utterances.

**Table 1:** General summary of the corpus resources used. Size is expressed in the total number of children's utterances.

**Corpus**	**Child's name**	**Begin**	**End**	**# of files**	Size (*CHI)
Antelmi	Camilla	2;2,06	3;4,09	7	1,892
Calambrone	Diana	1;8,05	2;6,13	9	2,196
Calambrone	Guglielmo	2;2,1	2;11,14	9	2,209
Calambrone	Martina	1;7,18	2;7,15	16	4,216
Calambrone	Raffaello	1;7,7	2;11,20	17	3,750
Calambrone	Rosa	1;7,13	3;3,23	21	7,409
Calambrone	Viola	1;11,16	2;10,3	10	2,667
Roma	Francesco	1;4,03	1;8,17	10	1,138
Tonelli	Elisa	1;10,04	2;1,23	8	1,090
Tonelli	Gregorio	1;7,17	2;0,29	8	1,121
Tonelli	Marco	1;5,04	2;5,24	19	6,787

The corpus analysis was performed alternating automatic and manual searches in order to address the empirical questions presented in (16) and (17). Our point of departure was to look for the spontaneous production of the three different particles *che, di*, and *se*. In the next section, we will consider them separately, also looking at the different types of embedding introduced by *che*. The results relative to the age of first occurrence of each particle will then be brought together in section *A General Overview: Comparing the First Uses of the Complementizer Particles*, to provide a comparative overview on the developmental course of the three particles. In section *Properties of Movement in the Extended Left-Periphery*, we will then turn to the analysis of movement constructions, looking for examples of multiple movements in the left-periphery and also for potential violations to the Q-Criterion.

## Complementizer Particles in Spontaneous Productions

We will first consider children's production of the particles *che, di*, and *se* that constitute the backbone of the CP-system. Our first step was to isolate children's use of each of these particles through an automated search using the *kwal* function on CLAN. Then, we manually went through the results and isolated all the occurrences in which *che, di, se* could be unambiguously classified as a complementizer particle. This allowed us to exclude other irrelevant forms, for example the homophonous preposition *di* and the 3rd person reflexive pronoun *se*. Finally, we further analyzed the results in order to consider the type of embedding introduced by the different particles. We will present the results by considering each particle in turn, beginning with the finite complementizer *che*.

### First Uses of the Particle *che*

The particle *che* in Italian is a versatile functional head performing different roles and occurring in different positions in the map of the left-periphery (Rizzi, 2013). Its core function may be identified in the expression of declarative force in embedded declarative clauses, but it also marks the CP-system of subject and object relatives, and of other kinds of main and embedded clauses.

Our automatic search revealed 508 occurrences of *che* in children's speech. Of those, 106 occurrences were classified as *not-clear*. We report the overall distribution of the remaining 402 over time. In order to capture the general longitudinal trend, we first divided the time-window covered by the 128 files in our corpus in 1-month intervals. Then, for each interval, we counted the total number of *che* produced by each child, excluding unclear cases. This procedure will be the same also for the other particles analyzed later. The results are summarized in Table 2, in which gray cells indicate the months for which at least one file is available. Gray cells provide a quick visual indication about the period covered by the transcriptions for each child: for example, Francesco's recordings start very early: the first gray cell is at 16 months. His transcriptions also end before the others, with the last gray cell at 20 months. When multiple recordings are taken within the same month, we collapsed the files together and report them within a single cell.

**Table 2:** Longitudinal production of *che* in children.

**Age in months**	**Children**											**Total**
	**Camilla**	**Diana**	**Elisa**	**Francesco**	**Gregorio**	**Guglielmo**	**Marco**	**Martina**	**Raffaello**	**Rosa**	**Viola**	
16				0								0
17				0			0					0
18				0			0					0
19				0	0		0	0	0	1		1
20		0		0	0		0	0				0
21					1		2	1	0	0		4
22		2	9		0		1	0	0	0		13
23		0	19				1	0	1	1	0	22
24		8			2		5		0	0	0	15
25		3	30				4	2	0	0	0	39
26	5		4			12				1		22
27						3		6	0			9
28	9					0		2	0	30	1	42
29		12				1		2	0	1	6	22
30	13	29							2	1		45
31						5		2	0	0	0	7
32									4		1	5
33	7					8			3	1		19
34						1				2	3	6
35	15					20			8	29		72
36										3		3
37	11									2		13
38												
39										10		10
40	34											34
Total	94	54	62	0	3	50	13	15	18	82	11	402

*Darker cells indicate months in which there is at least one transcription available, while white empty cells indicate months without any datapoint. Number in the gray cells represents the total number of occurrences per month. Non-Clear cases are excluded*.

Table 2 shows that all children, with the sole exception of Francesco, use the particle *che*. The absence of *che* in Francesco's transcription is most likely due to the fact that his recordings end much earlier than the others. For the remaining children, the table shows that in the initial period, between 16 and 20 months, the occurrences of *che* are scarce, with only a single occurrence over 16 files, found in Rosa's transcriptions. However, from months 21 to 26, most children start producing the particle *che* and by month 28 it is attested in the speech of all children, with the exclusion of Francesco for the reasons already discussed. It is also worth noticing that most of the transcriptions end at month 35, with the exception of Rosa and Camilla. Therefore, data in the time interval 36–40 months becomes more scattered, as shown by the paucity of the gray cells.

**Figure 1:**
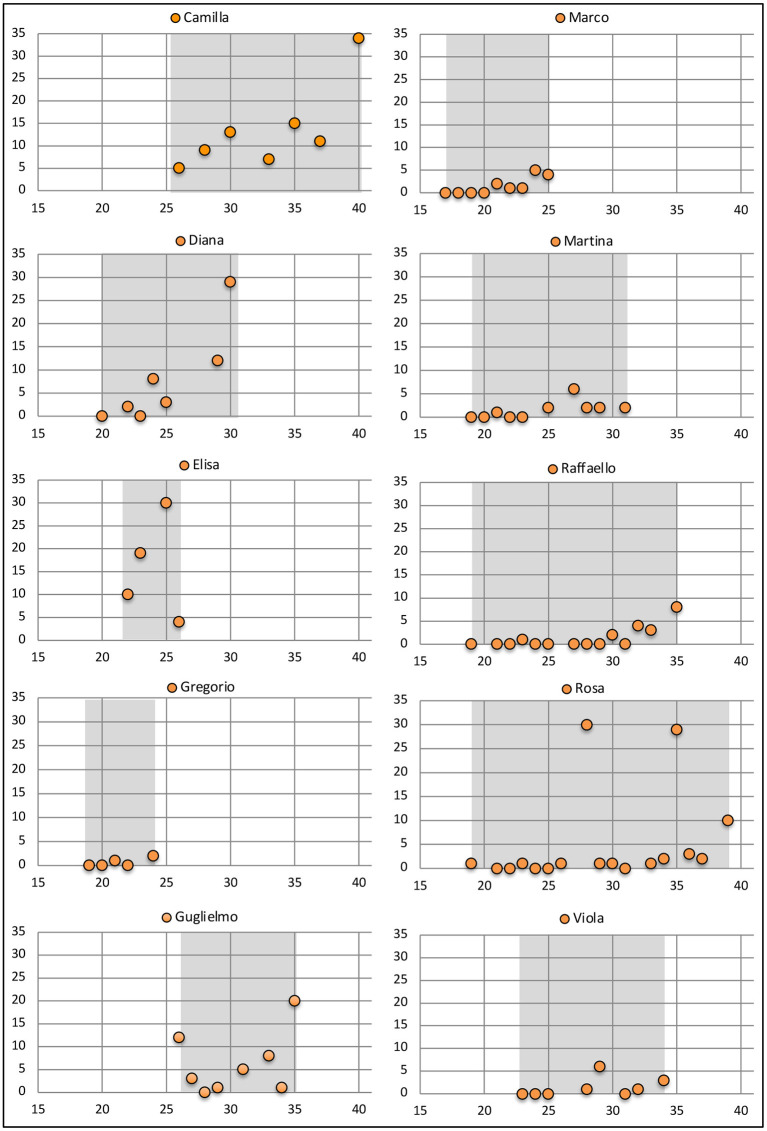
The number of *che* particles attested in the spontaneous production of each child by month. The X-axis reports the age in months, gray areas represent the period covered by individual transcriptions.

The longitudinal data showing production of the particle *che* for each child, excluding Francesco, is depicted graphically in Figure 1. The figure demonstrates quite clearly that most children have already started using this particle around the onset of the 2nd year. Fluctuations in the absolute number of particles produced, most evident in the two peaks observable in Rosa's transcriptions, may depend on the size of the single sessions and on the granularity of the sampling, that may vary.

**Figure 2:**
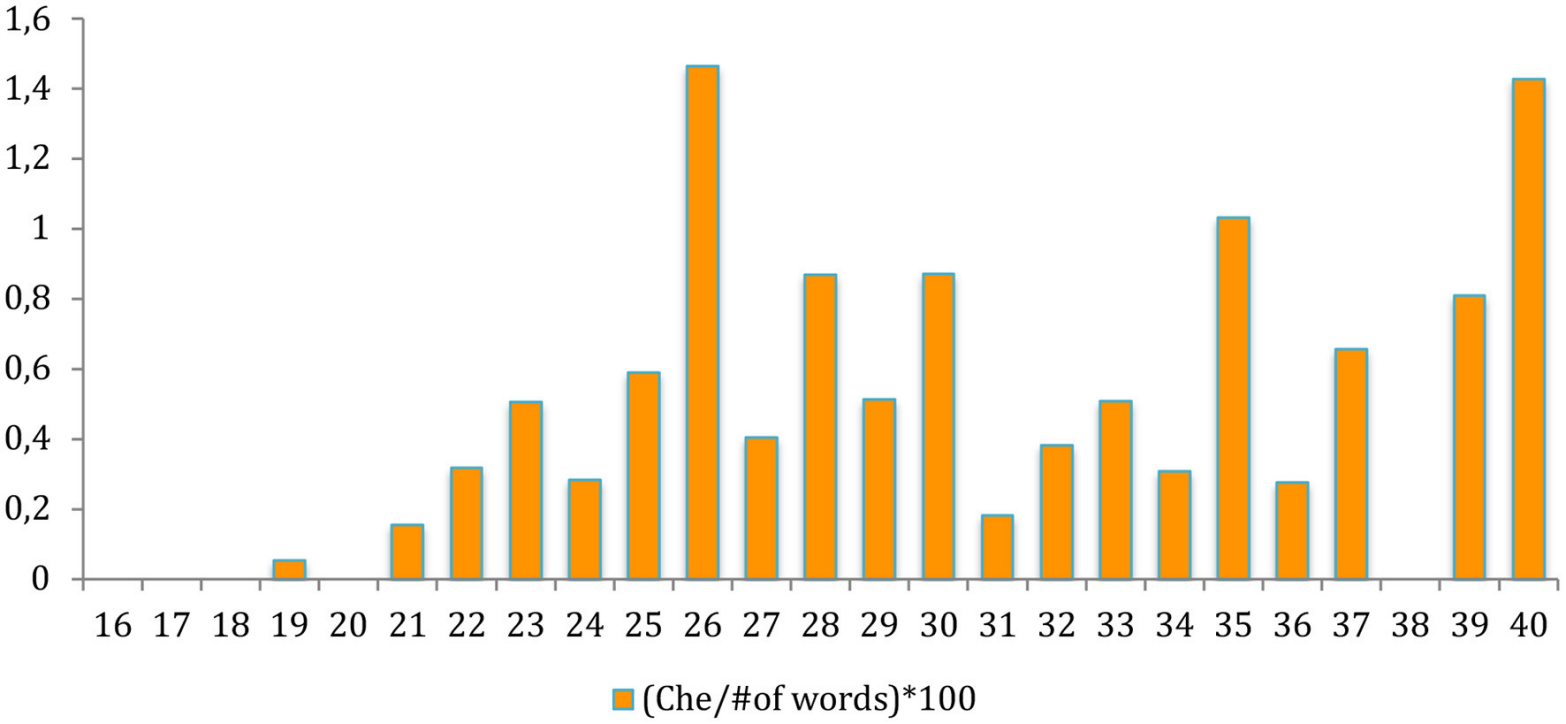
Ratio of the particle *che* over the total number of words uttered per month.

The fact that children start to use the particle *che* at around age 2 is also evident if we normalize the count in function of the total size of the transcriptions reporting the aggregated ratio *che*/number of words. The aggregate longitudinal ratio is plotted in Figure 2. Although there are fluctuations due to the available samples, evident in the “gap” at month 38, the plot also indicates that *che* becomes increasingly frequent between month 21 and 26, when the corpus reaches its maximal density.

Summarizing the results of this preliminary overview, it seems that by the middle of their 2nd year Italian-speaking children have already begun to produce the complementizer *che*, a prerequisite for the emergence of adult-like forms of embedding. Since this particle may serve a number of syntactic functions, not all related to subordination, a more fine-grained analysis is needed. We then went through each single instance and determined its syntactic function, to compare the incidence and the age of first appearance of the different syntactic structures.

### Clausal Embedding Introduced by the Finite Complementizer *che*

As a second step, we manually analyzed and classified each instance of *che*. Excluding the 106 unclear cases, the remaining 402 were sorted into 8 categories. We will briefly illustrate them in (18) below, using some real examples found in our corpus.


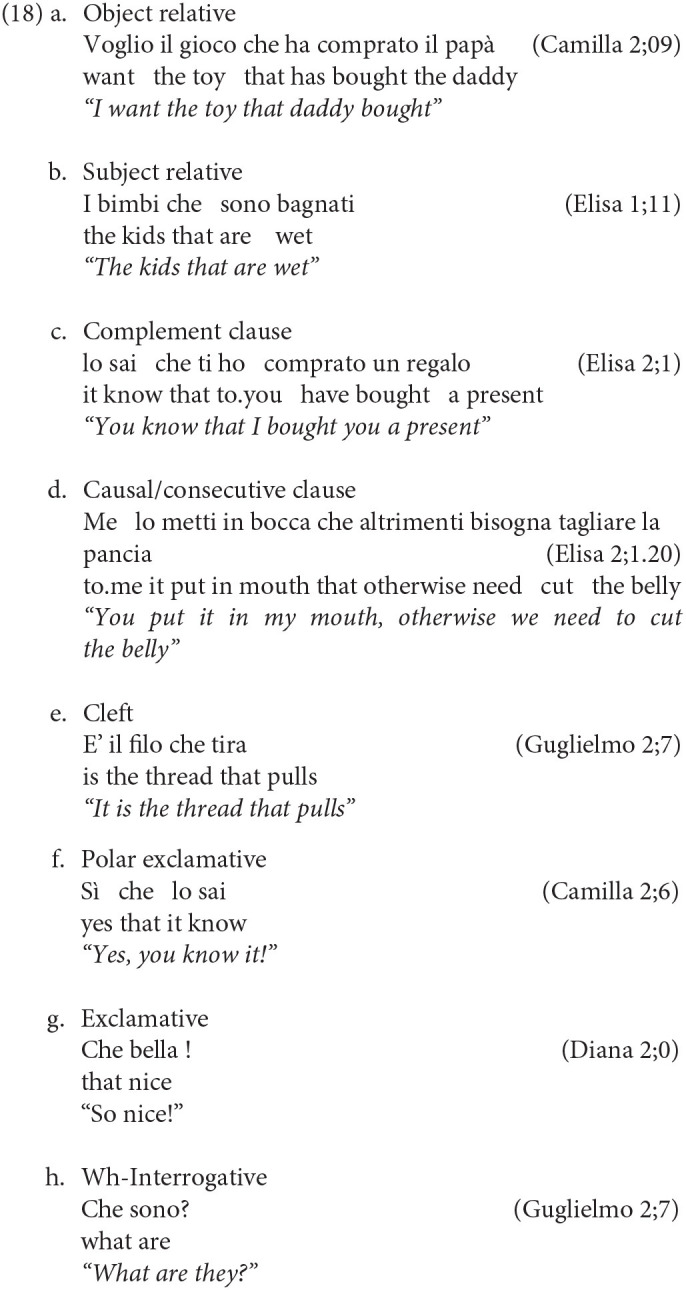


Consider first the examples in 18 (a-d). In these constructions the *che* particle clearly introduces a subordinate clause: subject or object relatives (18a-b), a complement clause (18c) or an adjunct in the form of a causal/consecutive clause (18d).

The categories in 18 (e-h) have been instead used to classify other uses of *che* that cannot be immediately reduced to clausal embedding. Sentence (18e) is the only cleft found in our corpus, so that, whether or not we consider clefts as illustrating a special case of subordination, this single example will not have an impact on the general results. Sentences (18f) and (18g) illustrate two different cases of exclamatives, where the particle *che* can be either preceded by a polar element (*si* “yes” or *no)* as in (18f) or be in sentence-initial position as in (18g). The two cases obviously differ in that *che* clearly is a C-particle in (18f), whereas it is a DP-internal wh-specifier of the exclamative phrase in (18g). Although a bi-clausal analysis is plausible for some of the constructions in (18e-f) (cleft: Belletti, 2015; polar exclamatives, Poletto and Zanuttini, 2013), they should be kept separate from the very clear cases of matrix/embedded subordination given in (18a-d). Finally, we also isolated sentences in which *che* is equivalent to *che cosa* exclamatives in (18h), in which *che* is a DP-internal specifier, not a head of the clausal spine.

The various uses of *che* attested for each child are reported in Table 3. A first observation that can be made is that all children in our corpus use *che* to introduce subordinate clauses, with the exception of Francesco and Gregorio, whose transcriptions end too early, and Viola. Of these subordinate clauses, in 12.9% of cases *che* is used with embedded complement clauses. Subject and object relatives cover instead, respectively, 21.0 and 5.0% of the data, with an additional 10.9% attributed to causal/consecutive adjunct clauses.

**Table 3:** Number of occurrences of *che* for each syntactic category per child and their aggregated proportion in total.

**Child**	**SR**	**OR**	**Complement**	**Causal**	**Cleft**	**Polar Excl**.	**Exclamatives**	**Wh-int**	**Tot**.
Camilla	29	8	17	11	0	11	5	13	94
Diana	7	4	5	7	0	7	13	11	54
Elisa	10	2	15	18	0	6	10	1	63
Francesco	0	0	0	0	0	0	0	0	0
Gregorio	0	0	0	0	0	0	3	0	3
Guglielmo	14	1	11	1	1	0	5	17	50
Marco	5	1	1	0	0	0	2	4	13
Martina	2	0	0	5	0	1	5	2	15
Raffaello	11	2	1	0	0	0	1	3	18
Rosa	7	2	2	2	0	0	2	67	82
Viola	0	0	0	0	0	0	8	3	11
Total	85	20	52	44	1	25	54	121	402
% on Total	21.0	5.0	12.9	10.9	0.2	6.2	13.4	30.1	

This distribution confirms that in about half of the cases, children use the particle *che* to introduce a finite subordinate clause. Therefore, in their third year of life, Italian children already make use of clausal embedding, correctly introducing them by means of the finite complementizer c*he*.

We are now in position to address one of our initial questions: is there any important difference in the timing of the appearance of relative clauses (18a-b) with respect to complement clauses (18c-d)? In order to answer this question, we further examined the transcriptions looking for the first occurrence of each type of embedding. The results are reported in Table 4, with the age of first appearance indicated in months.

**Table 4:** First occurrence of *che* indicated in months, by sentence type.

**Child**	**Type of Subordination**				**Pattern**
	**SR**	**OR**	**Complement**	**Causal**	
Diana	22	29	29	25	A. Rel > complement
Guglielmo	31	26	34	35	
Marco	24	24	25	–	
Rosa	36	33	39	39	
Camilla	29	28	28	–	B. Rel = complement
Raffaello	32	33	32	–	
Martina	27	–	–	27	
Elisa	23	25	22	–	C. complement > Rel
Francesco	–	–	–	–	n.a.
Gregorio	–	–	–	–	
Viola	–	–	–	–	

By looking at the first occurrences of the different types of subordinate clauses, Table 4 reveals that children do not conform to a single homogeneous pattern. Four children, namely Diana, Guglielmo, Marco, Rosa, produced their first relative clause before a complement/causal clause. In two of the children, Guglielmo and Rosa, an object relative was the first type of embedding. The opposite pattern, with a complement clause found before the first relative, was attested in the transcriptions of one child, Elisa. In the other three children, Camilla, Raffaello and Martina, the two types of embedding appeared at the same time. The remaining children did not produce any subordinate clauses in the available time-window, therefore they do not provide any data point.

In conclusion, there seems to be no uniformity across children in the order of first appearance and only a slight advantage of relatives over complement clauses can be observed in a subset of the transcriptions. The two types of embedding seem to blossom nearly together, suggesting that they could both appear as soon as the topmost functional projection, in this case ForceP, becomes available. The weak advantage of relatives could be due to their more free distribution, since their CP needs not encode grammatical traits specified by the embedding matrix verb, as suggested in Penner (1995).

### The Particle *Di*

We now turn to the complementizer *di* that occupies the lowest projection within the CP-system. This particle is at the nexus between the CP- and the IP-system and it introduces a non-finite control clause. In order to isolate it, we adopted the same procedure used with *che*: first, we ran an automatic search using the *kwal* command in CLAN and then we manually went through the occurrences to exclude the homophonous preposition *di*. Our search revealed that the use of *di* is much more limited than *che*, a fact that is not surprising given that its distribution is significantly more restricted, as it introduces only certain non-finite control clauses. Nineteen instances were found over 128 files, and they were all confined to the transcriptions of 5 children: Camilla, Diana, Elisa, Guglielmo and Marco. We report a few examples below:


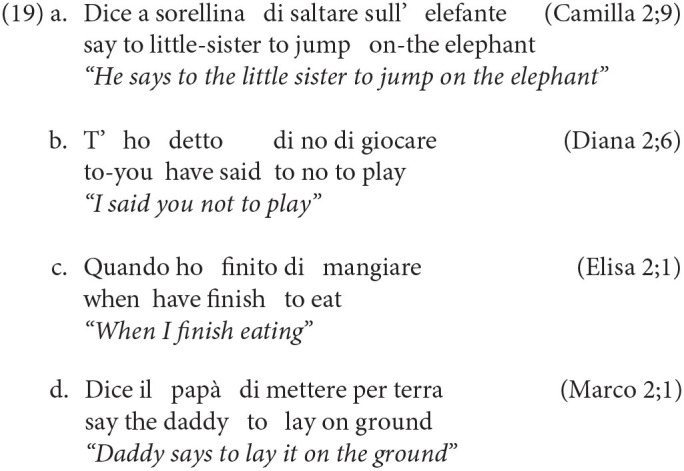


Again, to document the longitudinal trend, we divided the temporal continuum into 1-month intervals and report the occurrences for each child in Table 5 below.

**Table 5:** Longitudinal production of the non-finite complementizer *di* in children.

**Age in months**	**Children**											**Tot**.
	**Camilla**	**Diana**	**Elisa**	**Francesco**	**Gregorio**	**Guglielmo**	**Marco**	**Martina**	**Raffaello**	**Rosa**	**Viola**	
16				0								0
17				0			0					0
18				0			0					0
19				0	0		0	0	0	0		0
20		0		0	0		0	0				0
21					0		0	0	0	0		0
22		0	0		0		0	0	0	0		0
23		0	1		0		0	0	0	0	0	1
24		0			0		0		0	0	0	0
25		0	3				2	0	0	0	0	5
26	0		0			0				0		0
27						0		0	0			0
28	1					0		0	0	0	0	1
29		0				0		0	0	0	0	0
30	0	2							0	0		2
31						1		0	0	0	0	1
32									0		0	0
33	3					0			0	0		3
34						0				0	0	0
35	2					0			0	0		2
36										0		0
37	2									0		2
38												0
39										0		0
40	2											2
tot	10	2	4	0	0	1	2	0	0	0	0	19

*Darker cell indicate months in which there is at least one transcription available, while white empty cells indicate months without any datapoint. Number in the gray cells represents the total number of occurrences per month. Non-Clear cases are excluded*.

The results of this search show that the occurrences of *di* are rarer if compared with the early occurrences of *che*. At the same time, they also show that some children already use non-finite control clauses introduced by *di* around, or shortly after, their second birthday as we observed for the use finite subordination.

### The Particle *Se*

The third complementizer we considered is the particle *se* used to introduce indirect yes/no questions and conditional clauses. As with the other particles *che* and *di*, we ran an automated search in CLAN and then we looked over the results to manually exclude occurrences of the omophonous pronoun *se*. In total, our search produced 40 instances of *se* used as a complementizer. Fifteen other cases were excluded, since they were unclear. Again, the longitudinal prospect of individual productions is rendered in Table 6.

**Table 6:** Longitudinal production of the complementizer *se* in children.

**Age in months**	**Children**											**Total**
	**Camilla**	**Diana**	**Elisa**	**Francesco**	**Gregorio**	**Guglielmo**	**Marco**	**Martina**	**Raffaello**	**Rosa**	**Viola**	
16				0								
17				0			0					
18				0			0					
19				0	0		0	0	0	0		
20		0		0	0		0	0				
21					0		0	0	0	0		
22		0	0		0		0	0	0	0		
23		0	1		0		0	0	0	0	0	1
24		0			0		0		0	0	0	
25		0	1				1	0	0	0	2	4
26	1		0			0				0		1
27							0		0	0		
28	2						0	0	0	0	0	2
29		0				0		0	0	0	0	
30	0	7							0	0		7
31		0				3		0	0	0	0	3
32									0		0	
33	0					0			0	0		
34						1				1		2
35	2					1			9	1		13
36										0		
37	0									2		2
38												
39										1		1
40	4											4
Total	9	7	2	0	0	5	1	0	9	5	2	40

*Darker cell indicate months in which there is at least one transcription available, while white empty cells indicate months without any datapoint. Number in the gray cells represents the total number of occurrences per month. Non-Clear cases are excluded*.

The table above shows that the majority of children start using the particle *se* within the time-window covered by the transcriptions. The usual exceptions were Francesco and Gregorio because of their shorter and earlier recordings, and Martina. The earliest occurrence of *se* is found in Elisa's transcriptions, at 23 months. Notably, in the short time-window between 22 and 23 months, Elisa already presents all the complementizer's particle, that appear together at around the same time. In the other children, this particle appears before the third year: in fact, before 35 months, they all have produced at least one instance of *se*. We report below a few examples, showing the early use of *se* introducing different types of subordinate clauses, namely embedded interrogatives and conditionals.


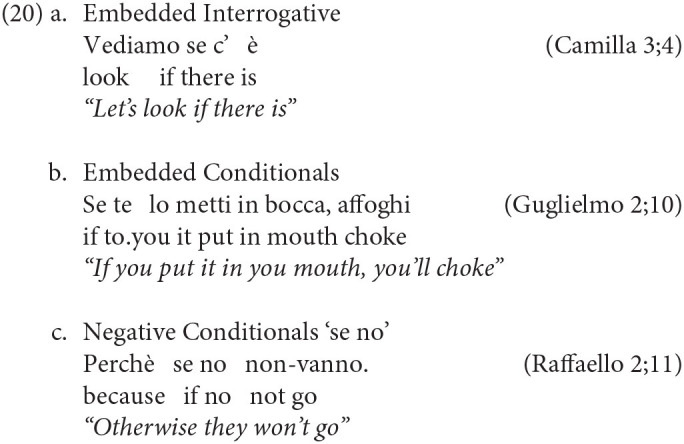


We found 4 embedded interrogatives (20a), 19 conditionals (20b) and 17 conditional expressions in the form of “se no” (20c).

At this point, having determined the onset of each particle, we can bring the findings together to draw a first sketch of the development of the CP-system through the appearance of the heads of functional projections that permit clausal embedding.

### A General Overview: Comparing the First Uses of the Complementizer Particles

We are now in a position to address our first question, concerning when the particles *che, se*, and *di* are attested in children's spontaneous speech. As a preliminary observation, our search revealed that between age 2 and 3 all CP-particles are already attested in many of the transcriptions. Moreover, in all children, their production conforms to the adult grammar. No misuse of *che, se*, or *di* was observed.

**Figure 3:**
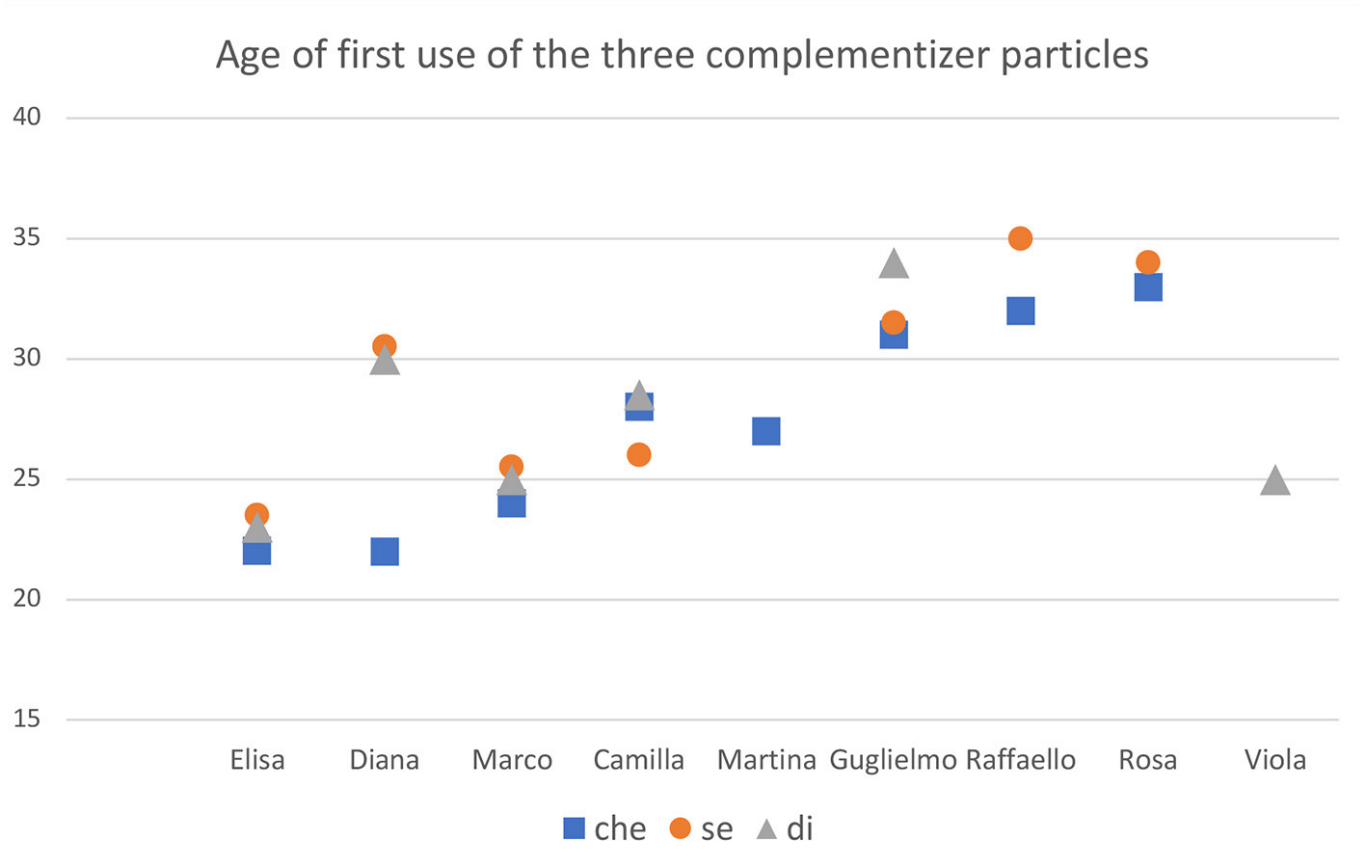
First use of *che, se* and *di* in the transcriptions of the 9 children. y-axis represents the age in months.

Turning now to the development of each particle and to their first documented occurrences, the more limited distribution of the particles *se* and *di* in the adult grammar has to be considered. These two particles also occur less often than the complementizer *che* in children's productions. Therefore, being less frequent, they are also more likely to slip through the mesh of a sparse sampling. Conversely, the first occurrences of *che* have a higher probability to be captured. On the basis of frequency-based considerations alone, we could then expect that *che* will emerge earlier in the corpus analysis, since a comparison between the first attestation of the three complementizers is strongly biased in favor of *che*. The pattern that we found comparing and merging the results presented in previous sections is not entirely consistent with this expectation. We illustrate it visually by reporting in Figure 3 the age of first use of each particle. In the case of *che*, we only report occurrences in which it was used to introduce a subordinate structure. Figure 3 shows that only in the cases of Diana, Martina and Raffaello the particle *che* clearly precedes the other two and it is attested at least a full month earlier: in Diana, this particle preceded the other two of a few months; in Martina, it is the only one attested; in Raffaello it appears well-before *se*, while *di* is still absent.

This trend, however, is not predominant across the transcriptions in our corpus. Figure 3 shows that in the majority of children the particle *che* either appears at almost the same time of one or both the other two particles (Elisa, Marco, Guglielmo, Rosa) or even later as in the case of Camilla and Viola (it is still absent in this latter child). Thus, despite the higher overall frequency of *che*, its advantage in terms of its first occurrence is rather weak and in four children, Elisa, Marco, Camilla and Guglielmo, the three particles appear at around the same time. In the case of Elisa, they all appear before the 2nd year and all within a 1-month interval, between 22 and 23 months. In Elisa's grammar, the CP-system thus stabilizes very early. Within the margins of individual variations, the same also happens in Marco, Camilla and Guglielmo, where we still observe the appearance of the three particles in a short time-interval. Raffaello and Rosa do not show any attested occurrence of *di*, but have *che* and *se* appearing in rapid sequence.

Our data suggest that the temporal advantage of *che* over the other two particles is not generalized. This is consistent with the conclusion arrived at in Friedmann et al. (2020) on the acquisition of modern Hebrew, where different types of clausal embedding are attested at once. Speculating on this observation, it is then possible that all forms of clausal embedding could be related to the emergence of a single property. In particular, the availability of the topmost clausal projection, ForceP, could provide the impetus for the emergence of embedded clauses of different kinds. Assuming that the selection of finite embedded clauses is categorially uniform and that all verbs selecting a finite complement select a ForceP under sisterhood (Chomsky, 1995), all kinds of finite embedding would thus need to establish a link with the topmost projection ForceP, where the appropriate grammatical trait is encoded so to satisfy the requirements of the matrix embedding verb. The consequence of this is that other types of embedded clauses, such as the conditional clauses introduced by *se*, are only possible if ForceP is already available in the early clausal structure. Thus, we can consider the appearance of *che* as a signal that ForceP can be projected. From this moment on, other different types of embedded clauses would emerge at the same time, or shortly after. We thus expect that *che* could either slightly precede *se* and *di* (with frequency being a confounding factor), or that the three particles become simultaneously accessible upon the availability of ForceP. We return to this at the end of the general discussion, when we will present the hypothesis that the clausal structure undergoes a stepwise maturational growth.

## Properties of Movement in the Extended Left-Periphery

Beside the functional projections that are headed by an overt particle, the extended CP of Italian also features a series of syntactic positions that can host A'-movement. Looking into these constructions could also provide important indications on the early structure of the CP-system. In what follows, we will examine in detail some properties of the Wh-questions attested in our corpus.

The first property we will consider is children's compliance with obligatory inversion, triggered by Wh-elements moved into the Q/FocP position. As discussed earlier, this operation is needed in order to satisfy the Q-Criterion, requiring a local spec-head configuration between the wh- and the verbal head. In contrast, *why-*questions where the wh-element is base generated in IntP, do not require inversion (Rizzi, 2001). The important consequence is that inversion is activated or not depending on the syntactic position of the Wh-constituent. Therefore, if children distinguish between positions requiring inversion (Q/Foc) vs. positions which do not (IntP), we should find a difference in the rate of inversion between *why-*questions as commpared to other wh-questions.

A second property that could be revealing about the stratification of the early CP-system is the possibility for multiple movements to the left-periphery. The structure of the Italian CP allows for the simultaneous occurrence of Wh- and topic movement, due to the availability of Topic projections above Q/FocP. In fact, sentences where a topic precedes a wh-pronoun are perfectly acceptable in the adult grammar of Italian and have also been reported in the spontaneous productions of the three European Portuguese children studied by Soares (e.g., *O gato, onde está* “the cat, where is it? p. 290 Soares, 2006).

In order to look for inversion and multiple movements, we are now primarily interested in sentences containing an interrogative pronoun. Therefore, we first isolated all the wh-sentences in our corpus. To do this, we ran an automated search looking for Wh-pronouns in Italian (*cosa, chi, dove, quale, come, quando, quant-o,-e, -a, -i, perché*) and 955 wh-elements were isolated within the transcriptions of 10 children^3^. From those, 124 occurrences were unclear and excluded. Then, the remaining 831 occurrences were manually examined and classified so as to distinguish between fragments, matrix and embedded questions. This latter class includes both (i) full bi-clausal constructions with a subordinate clause embedded under an overt matrix and also (ii) subordinate clauses uttered in isolation, without the matrix. A further class was also included to account for the occurrence of wh's in their non-interrogative, exclamative use. We used 6 categories in total, illustrating them as usual by reporting some real examples from our corpus.


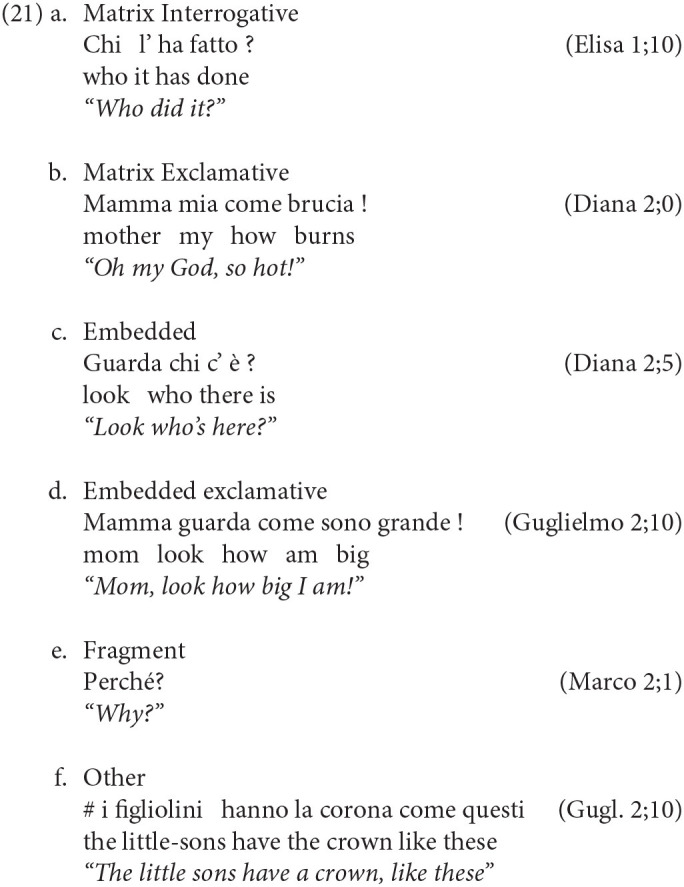


Clearly, the matrix wh's in (21a) are the most interesting construction to determine the subject-verb inversion rate. Looking at our corpus, the search confirmed that matrix wh's interrogatives are very frequent at this age, with the highest number among the other categories reported in Table 7^4^.

**Table 7:** Total number of wh-sentences classified for the categories in (21).

**Matrix Interr.**	**Matrix Excl.**	**Embedded**	**Embedded Excl.**	**Fragment**	**Other**	**Total**
378	11	184	3	237	18	831

In order to be able to determine the rate of subject-verb inversion, we also needed to distinguish between the different types of wh-elements. Thus, we went through all 378 matrix wh-questions, classifying them according to the wh pronoun. We also kept occurrences of *che* and *chi* separate, depending on their grammatical function. The results are reported in Table 8.

**Table 8:** Matrix wh-questions by wh- pronouns.

**Child**	**Chi** ***who***			**Cosa** ***what***		**Come *how***	**Dove *where***	**Perchè *why***	**Quant-*how much***	**Quando *when***	**Tot**
	**Subj.**	**Obj.**	**Other**	**Obj.**	**Subj.**						
Diana	6	0	0	4	0	6	10	0	2	0	28
Elisa	4	0	2	5	1	9	9	4	1	1	36
Francesco											
Gregorio											
Guglielmo	13			9	1	5	15	40			83
Marco	27		1	9	3	3	20	2			65
Martina	3			2				2			7
Raffaello	7	2	3	19	2	2	5	2		3	45
Rosa	86	1		4	7	5	7	1			111
Viola	3										3
Tot.	149	3	6	52	14	30	66	51	3	4	378

At this point, with this general picture at hand, we can move on and consider inversion and multiple movements in turn.

### Inversion and the Q-Criterion

We will look first at children's sensitivity to the different positions of the interrogative pronoun, as it could be determined by looking at the inversion between the subject and the verb. As we said, a distinctive grammatical property of the wh-elements in Q/Foc is that they require an overt spec-head relation between the Wh- and finite verb/auxiliary. On the other end, *why* questions behave differently since they do not trigger inversion. In fact, other constituents - including the subject-, are free to occur between *why* and the verb.

In order to analyse the rate of subject-verb inversion in questions, we further analyzed our set of 378 matrix wh-questions. We filtered out subject wh-questions and, for the remaining ones, we isolated sentences that presented an overt subject. As a result, we obtained 69 wh-interrogatives in which the subject could potentially intervene between the Wh- and the verb, in violation of the Q-Criterion.

Then, for each interrogative pronoun, we counted the occurrences in which the subject actually intervened between the wh- and the finite verb/auxiliary. The results are illustrated in Table 9.

**Table 9:** Number of matrix wh-questions presenting the overt subject occurring between the interrogative pronoun and the verb.

**Child**	**Chi_**Obj**_ “who”**	**Chi_**Other**_ “who”**	**Come “how”**	**Cosa_**Obj**_ “what”**	**Dove “where”**	**Quanto “how much”**	**Perchè “why”**
Diana			0/3	0/1	0/4		
Elisa			0/3	0/3	0/8	0/1	1/2
Francesco							
Gregorio							
Guglielmo			0/2	0/3	0/6		6/9
Marco		0/1	0/1	0/1	0/6		
Martina				0/2			
Raffaello	0/2		0/1	0/1	0/2		
Rosa	0/1		0/1	0/2	0/3		
Viola							
Total	0/3 0%	0/1 0%	0/11 0%	0/13 0%	0/29 0%	0/1 0%	7/11 63.6%

Table 9 shows that in matrix wh-questions the subject never precedes the verb, with the exception of sentences with *perchè/why*. In fact, we found only 7 sentences in which the subject breaks in the Wh-/Verb cluster^5^. Importantly, they are all *why* questions and they constitute no violation of the Q-Criterion. All the examples, mostly found in Guglielmo's transcriptions, are reported below:


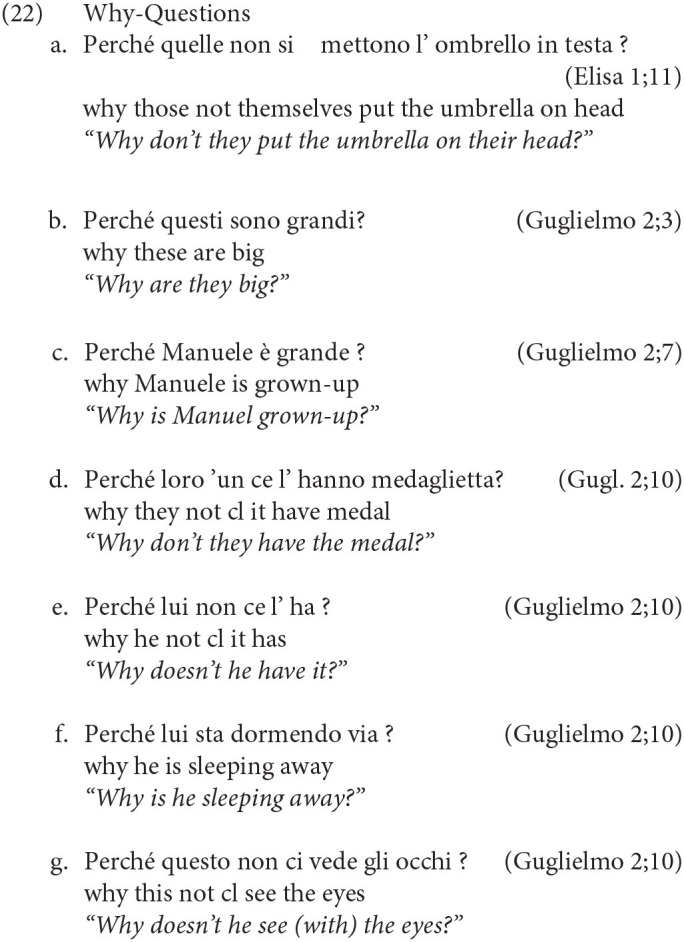


The analysis of the subject/verb inversion rate thus shows very clearly that young children already differentiate between the properties of distinct wh-elements. We take this selective compliance to the Q-Criterion as a first indication that 2-year-olds are already sensitive to the syntactic requirements associated with the functional projections in the CP-system.

### Multiple Movements to the Left-Periphery: Pre-focal Topics

After isolating matrix wh-questions in children's spontaneous speech, we performed a further analysis, looking for sentences in which wh-movement could have co-occurred with topicalization in the left-periphery. If attested, these sentences would indicate that both the Q/Foc position and a Topic position are activated together. Thus, providing evidence in favor of an early CP-system already populated by a cluster of different projections.

A pattern very robustly attested cross-linguistically is the possibility of a complex left-periphery consisting of a topic followed by a wh- element. This can involve both a regular wh-element occurring in Spec Foc/Q, and an element like *why*, occurring in the Spec of the higher position Int. This pattern is also possible in adult Italian, as illustrated by sentences (23) and (24). Consider first (23), in which the subject occupies a high topic position. This topic position is above Q/Foc, as the structural representation in (23′) shows.


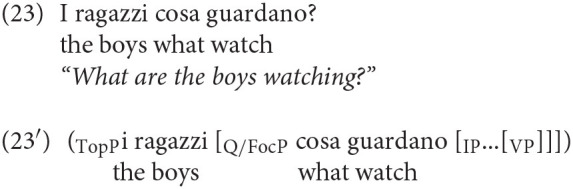


Similarly, in (25) the subject is also in a high topic position above *why*. This latter element is hosted in IntP (25'):


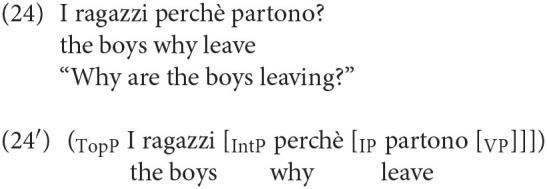


A similar instance of a complex CP, widely attested in adult Italian, is one in which the wh-element is preceded by a vocative phrase, occupying another dedicated left-peripheral position (see Moro, 2003 on vocative phrases):


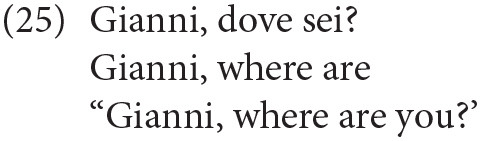


The complex sentences (23), (24), and (25) are perfectly natural in Italian and, very interestingly, we found that sentences of the same kind are clearly attested in children's productions. We found 20 examples in which the Wh- is preceded by a Topic or a Vocative phrase hosting the subject. Thus, if we exclude sentences in which the wh-element is the subject, for which no higher topic is attested, the incidence of these 20 examples over the remaining 215 wh-interrogatives is a non-negligible 9.3%. We report some examples in (26) below:


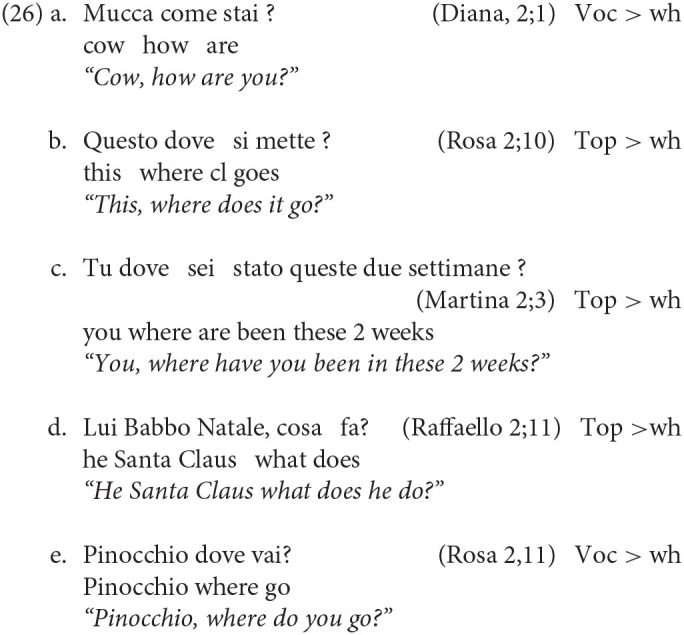


This shows that non-canonical sentences presenting the pattern *S*_*Top*_ *Wh*_*Q*_ *V* (or *Voc Wh V*) are attested already in very young Italian children, providing evidence in support of an already layered left-periphery. The very presence of these complex constructions shed new light on the early availability of Topic projection(s) in children's clause structure. Since this type of sentences is still poorly documented in early Italian (for French, see de Cat, 2007), it would be of great interest to establish, through future studies employing a more extensive manual search, what the distribution of overt topics is in the early speech of Italian children.

## General Discussion

By analyzing the transcriptions of a group of Italian children, we aimed at documenting the development of their early CP-system following two pathways. The first consisted in tracking down the early occurrences of the complementizer particles found in their speech and the emergence of embedded clauses. Our corpus analysis showed that the complementizer *che* is robustly attested early in the 3rd year of life and that also *di* (marking control infinitives) and *se* (marking embedded questions) are available at the same stage, or immediately after. The individual variation on how early embedded structures appear is remarkable, but from our search it emerged that Italian children start using sentential embedding soon after their second birthday, with many of them already employing the whole inventory of complementizer particles.

We also looked at the age of first appearance of the different types of finite embedded clauses introduced by the complementizer *che* in order to assess whether relative clauses are the first type of embedding available to the child, as observed in other languages by Penner (1995) and Armon-Lotem (2005). This advantage was accounted for by capitalizing on the absence of C-selection on relative clauses. Children could then be able to generate relatives before complement clauses since the former do not require lexical access to the grammatical traits of the embedding verb. Returning to the results of our analysis, the conclusion that relative clauses precede other types of clausal complement is only weakly supported in Italian, and this pattern was observed in only half of our children. The opposite direction was instead found in one child, Elisa, while in other three children the two different types of embedding appear at the same time.

Beside the analysis of complementation, we also extended our research in another direction, looking at the properties of early wh-questions. Wh-interrogatives also activate the CP-system and are informative on children's sensitivity to the different syntactic requirements on specific functional projections. Our search showed that, from very early on, children selectively perform I-to-C movement of the finite verb but only if the wh- element is moved in Q/FocP. By cotrast, inversion is not required with *why*/*perché* questions, much as in adult grammars. Our data are in line with similar results reported in Guasti (2000). Moreover, the early sensitivity to the specific properties distinguishing *perché* from other wh-elements in Italian also concurs with Thornton's (2008) important observation that some English-speaking children behave similarly, even though this distinction is not grammaticalized in adult English.

Our analysis did not only show an overall adult-like behavior with the properties of the single overt functional heads in the CP-system, but it also provided evidence for a layered structure in which different positions can be simultaneously realized according to the adult hierarchical order. In view of the many discourse-pragmatic constraints that should be satisfied, the relevant constructions can be hard to find and to elicit in young children. However, wh-questions targeting the focus position in main clauses are common enough to look for the co-occurrence of focus and topic movement. By looking at wh- sentences, we found that children were also able to project a layered CP by realizing both a Topic (or the Vocative position akin to TopP) and a Q/Foc or IntP position together. These complex sentences did not only indicate a refined syntactic competence supporting a layered CP-system, but they also witness a clear sensitivity to the interface between syntax and discourse pragmatics, as uses of topics were invariably appropriate to the particular discourse conditions.

Undoubtedly, the results presented here only provide a first sketch of the early CP-system and more needs to be found by combining targeted experimental investigations with additional corpus studies. In this respect, the collection of supplementary resources with a higher density of diary recordings could help to better define the developmental pattern of less-frequent constructions, overcoming some of the limitations of the present study and allowing to test specific research hypotheses also through the use of inferential statistics.

In concluding the paper we wish to tentatively consider how our results could relate with a recent developmental hypothesis that specifically targets the grammatical growth of the CP-system. According to the proposal presented in Friedmann et al. (2020) that we will briefly discuss next, the initial clause-structure available to the child could be a reduced version of the adult structure. This idea is conceptually in line with previous suggestions that the acquisition of syntax proceeds incrementally starting from the lowest portion of the tree (e.g., the VP node only, Lebeaux, 1988; Platzack, 1990; Radford, 1990; a single XP above VP, Clahsen et al., 1993/1994). It is also compatible with the proposal that, once available, higher layers can be “truncated” (Rizzi, 1993/94) leading to optionality in the realization of the topmost portion of the clause.

### A Stepwise Maturation of the CP-System

In a very recent proposal, Friedmann et al., 2020 put forth the “Growing Trees” proposal by which the left-periphery grows in the child's mind following steps that are consistent with the articulated map postulated in cartographic research. While our research on the early left-periphery in Italian was not conceived on the basis of this kind of developmental hypothesis, we think it is interesting to briefly investigate the compatibility of our findings with it.

Looking at both spontaneous productions and at the results of a repetition experiment with Hebrew-speaking children, Friedmann et al., 2020 advanced the proposal that the articulated structure of the CP does not emerge at once, but that it instead develops into three successive stages: the first shows mastery of the basic clausal structure (the IP) with no manifestation of the left-periphery: the second stage shows knowledge of the lower portion of the left-periphery including, among other positions, the landing site of wh-movement, and the third stage manifests mastery of the higher part of the left- the target of selection from higher selectors, which makes different kinds of embeddings possible. In this sense, the syntactic tree “grows” from a total absence, to a partial specification, and then to a complete specification of the CP-system.

This model was supported by the fact that after the first stage in which no peripheral construction was manifested, at the second stage of development, children acquiring Hebrew could ask *wh-*questions by productively fronting interrogative pronouns like *who, what, where*, etc. At the same age, they were not able yet to produce embedded clauses (declaratives or relatives), nor *why*-questions, nor topicalized structures. Capitalizing on the cartographic map of the CP-system, Friedmann et al. (2020) observed that the structures appearing in the following (third) stage have the property in common that they need to activate projections in the highest layer of the left-periphery, ForceP (for relatives, Force is required to hosts the relative operator; for embedded declaratives, Force is required in order to satisfy selectional properties from higher selectors), IntP (for *why* questions) and TopP, respectively.

In this model, at the second stage, children would only have a partial representation of the left-periphery, projected up to Q/FocP. It is only in a later third stage that the CP-system grows so to include higher positions, and the full adult structure.

If the same reasoning is applied to Italian, we would also expect a division of the CP-system into zones that would become available sequentially: after a first stage with no manifestation of the CP-system, we would expect a second stage with the lowest portion of the CP, up to Q/FocP, and then, successively, a third stage, with the upper part of the CP including IntP and ForceP. The second and third stages, and the functional projections available in them, can be represented as in the Table 10. Remember that stage I corresponds to a developmental stage in which the left-periphery is totally absent and the lower IP layer can be still in the process of growth.

**Table 10:** Stages II and III in the development of the left-periphery, according to the growing trees proposal; Stage II and III follow in order Stage I, that corresponds to the availability of lower IP-internal projections.

**ForceP**	**IntP**	**TopP**	**Q/FocP**	**ModP**	**FinP**
***Che***	***Perché/‘why’***		***Cosa/What***		
	Stage III		Stage II		

According to this model, constituent questions targeting the Q/FocP layer should be available before *why*-questions, which would only be manifested at stage III. In this later stage, *why-*questions and clausal embedding requiring the projection of ForceP would appear together. The particle *che*, an overt manifestation of ForceP, would then appear together with *why*, following other wh-questions. Other lower complementizer heads, as *se* and *di*, are also predicted to be available only at stage III since they also require a link with ForceP in order to satisfy the C-selection properties of the embedding verb. In Figure 3, we showed that in many children the three particles indeed appear around the same time.

In order to check if the overall developmental pattern presented in Table 10 is supported by the spontaneous production of our Italian-speaking children, we combined different data points relative to the three constructions that are relevant for evaluating the growing-trees hypothesis:


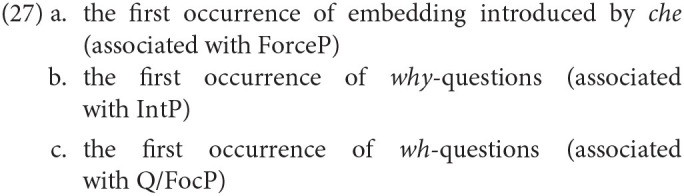


**Figure 4:**
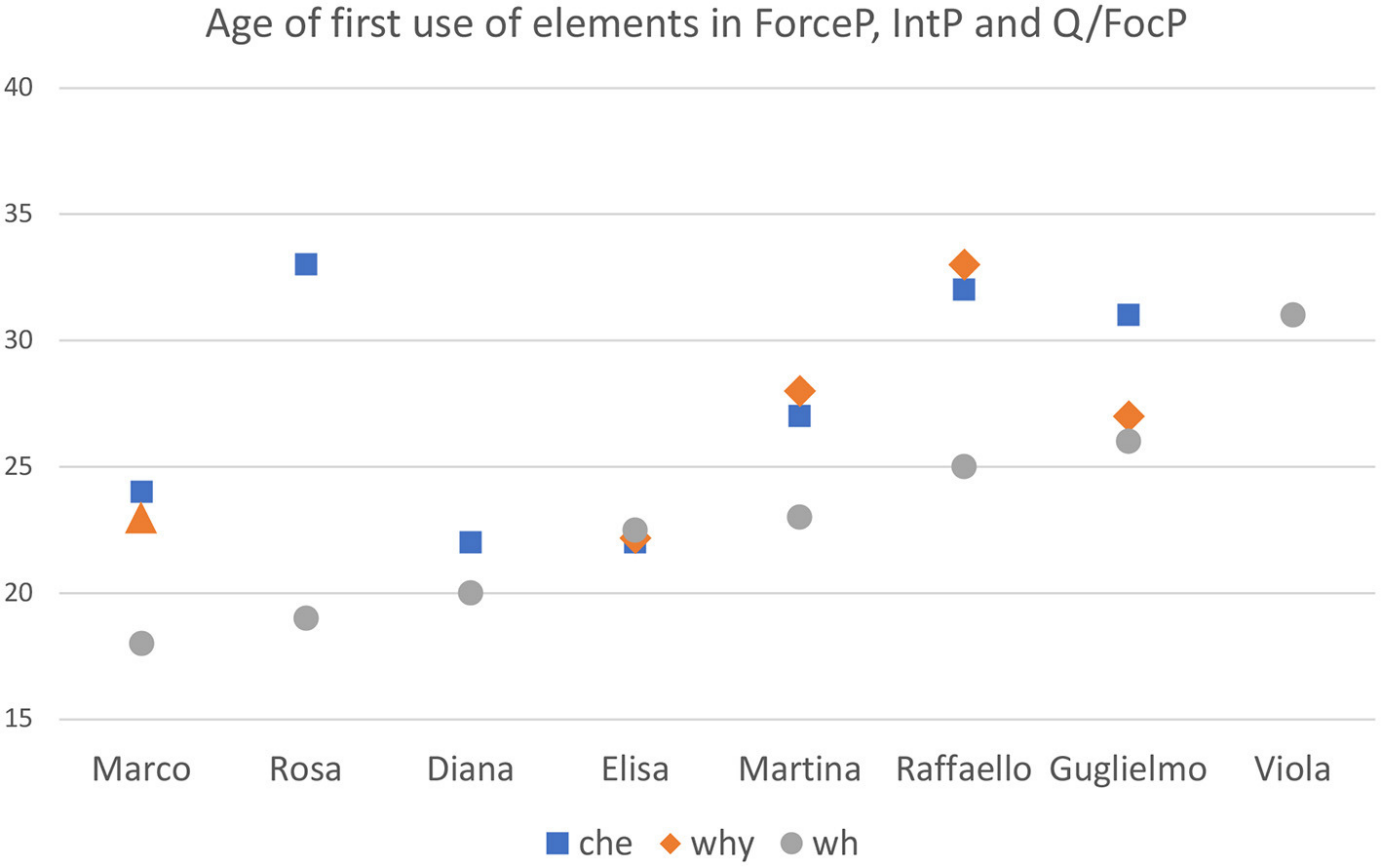
Age of first occurrence of: **(A)** finite embedding introduced by *che*; **(B)** *why* questions; **(C)**. wh-questions. Data are reported for each child, with the exclusion of Francesco and Gregorio, whose transcriptions end too early and Camilla (see footnote 3).

With respect to the constructions in (27), the prediction is that sentences in (27a-b) should never be attested before (27c), since their development is contingent upon the availability of the lower portion of the three. To evaluate this, we confront the age of first occurrence of each of the constructions in (27). The results are plotted in Figure 4. For each child, we indicated the month in which the specific construction was attested for the first time.

Figure 4 shows that wh-questions are the earliest to be attested in relation to the other types of sentences in (27a-c) and this pattern is consistent across all children. Also notice that, if only a subset of the constructions in (27) is produced, this invariably includes wh-questions, expected at the earliest stage in which the CP structure is manifested (stage II). In general, constructions that belong to the successive stage involving the growth of the upper part of the CP (stage III) are absent (as in Rosa, Diana, Viola) or delayed if compared with the emergence of wh-questions (as in Marco, Martina, Raffaello, Guglielmo).

Only in the case of Elisa, they all appear at the same time. This child showed an already fully-fledged CP before the end of the 2nd year – remember that she also produced all the CP particles – thus no developmental effect is visible in this case: Elisa already made it to stage III before her second birthday. This should come as no surprise, given the important individual variation in the speed of development: whereas the transition from one stage to the next may occur at very different ages in individual children, what the Growing Trees approach expects is that the sequence of stages will not be violated in the developmental path of a particular child, and this is indeed what we observe in the data.

## Conclusion

Cartographic research showed that the CP-system should be split into a sequence of functional elements (Rizzi, 1997), much as the IP system (Cinque, 1999). These findings raise questions for language acquisition: how and when are these complex configurations acquired by the learner? In this paper we tried to address these questions for the development of the CP-system in Italian.

The CP-system clearly is part of the child's grammar from the beginning of the 3rd year of life, or even earlier. Moreover, it can be confirmed that the child has access to the fine details of the CP structure. On the one hand, the child is sensitive to the position of occurrence of the wh-element in the fine structure of the CP, differentiating the case of the landing site of ordinary wh-movement, Foc/Q (for elements like *who, what, where*, etc.), and the case of *why*, base-generated in the higher position Int. In the adult grammar, ordinary wh-elements require inversion, whereas *why* does not, a property derived from the criterial approach in the analysis we have adopted. Such a different behavior of ordinary wh-elements and *why* is already reflected in the early productions we have examined. On the other hand, the corpus study provided clear evidence that young children are already able to produce complex CP systems with the co-occurrence of distinct elements in the CP space: this was shown by productions involving a topic followed by the wh-element.

In the final part of the paper we also discussed the implications of our results for the Growing Trees approach (Friedmann et al., 2020). This approach assumes that the CP-system develops in three successive steps: the first in which the CP-system is absent; the second, which involves the lower zone of the left-periphery, with the landing site for wh-movement; and the third, involving the upper left-peripheral zone, hence specifying the position of occurrence of *why* (in Int) and the Force position expressed by *che*. We observed, in line with the Growing Trees approach, that ordinary wh-movement is systematically attested earlier than *why* questions and embedded declaratives introduced by *che*. We very much hope that our preliminary results will trigger more corpus-based and experimental research to make advances on the acquisition of the complex structural configurations uncovered in cartographic research.

## Data Availability Statement

Publicly available datasets were analyzed in this study. This data can be found at: childes.talkbank.org.

## Ethics Statement

Ethical review and approval was not required for the study on human participants in accordance with the local legislation and institutional requirements. Written informed consent from the participants' legal guardian/next of kin was not required to participate in this study in accordance with the national legislation and the institutional requirements.

## Author Contributions

VM and LR collaborated together on all stages of the present work. Both authors contributed to the article and approved the submitted version.

## Conflict of Interest

The authors declare that the research was conducted in the absence of any commercial or financial relationships that could be construed as a potential conflict of interest.

## Footnotes

^1^The map differs from the map in Rizzi and Bocci (2017) in that it does not specify the very low (post-focal) topic position, a marked position which Italian permits, but which will not play a role in our analysis. It is typically observed following correctively-focused fronted constituents, unattested in early spontaneous speech. Our analysis also does not include the position Mod in the lower part of the left-periphery, dedicated to attracting highlighted adverbials.

^2^In adult speakers the representation in (5) is revealed by sequences of contrasts in grammaticality, carefully assembled. Although judgments are sharp, as in the examples we reported in (2-4), sentences of this kind are infrequent also in adult production. Therefore, a direct comparison between the adult structure uncovered by target grammaticality judgments and the initial structure hypothesized by children would necessarily require a controlled experimental set up.

^3^We excluded the files from Camilla. The reason is that we realized that in Camilla's transcriptions accented characters are not correctly encoded. Thus, for example, the 3^rd^ person form of the verb “to be” è is substituted by the symbol #, which is used in the CHAT format to indicate pauses. This will be problematic once we look at the position of the Wh-element in relation to the finite verb or the auxiliary.

^4^Matrix wh- appear generally earlier than full bi-clausal sentences. Examples like (21c) are attested only after matrix wh's. For instance, the first full form of embedded interrogative is found in Elisa's corpus at 1;11, when matrix wh's were already attested: As in the case of lower CP heads, embedded wh's would also require a link to ForceP, to satisfy C-selection.

^5^We found only a single example in which an argumental wh- does not immediately precedes the lexical verb: But this seems to be a subject question, with the wh-subject appearing *in situ* in the postverbal subject position, a non-target configuration in Italian. In any event, assuming it is a subject question, this example is not relevant for determining adherence to the Q-criterion in child grammar.
